# Expression Pattern of ERF Gene Family under Multiple Abiotic Stresses in *Populus simonii × P. nigra*

**DOI:** 10.3389/fpls.2017.00181

**Published:** 2017-02-20

**Authors:** Wenjing Yao, Xuemei Zhang, Boru Zhou, Kai Zhao, Renhua Li, Tingbo Jiang

**Affiliations:** ^1^State Key Laboratory of Tree Genetics and Breeding, Northeast Forestry UniversityHarbin, China; ^2^Northeast Institute of Geography and Agroecology, Chinese Academy of SciencesHarbin, China

**Keywords:** poplar, ERF transcription factor, abiotic stresses, *ERF76* gene, expression pattern

## Abstract

Identification of gene expression patterns of key genes across multiple abiotic stresses is critical for mechanistic understanding of stress resistance in plant. In the present study, we identified differentially expressed genes (DEGs) in di-haploid *Populus simonii × P. nigra* under respective stresses of NaCl, KCl, CdCl_2,_ and PEG. On the basis of RNA-Seq, we detected 247 DEGs that are shared by the four stresses in wild type poplar, and mRNA abundance of the DEGs were validated in transgenic poplar overexpressing *ERF76* gene by RNA-Seq and RT-qPCR. Results from gene ontology analysis indicated that these genes are enriched in significant pathways, such as phenylpropanoid biosynthesis, phenylalanine metabolism, starch and sucrose metabolism, and plant hormone signal transduction. Ethylene response factor (ERF) gene family plays significant role in plant abiotic stress responses. We also investigated expression pattern of ERF gene family under the four stresses. The ERFs and DEGs share similar expression pattern across the four stresses. The transgenic poplar is superior to WT in morphologic, physiological and biochemical traits, which demonstrated the *ERF76* gene plays a significant role in stress resistance. These studies will give a rise in understanding the stress response mechanisms in poplar.

## Introduction

As the first sequenced woody plant, poplar is a model species for mechanistic understanding of stress resistance, as it’s provided with rapid growth process, easy vegetative propagation and small genome size in perennial plants (Tuskan et al., 2006), which facilitates genome-wide analysis of gene families related to environmental stress, such as HD-ZIP (homeodomain-leucine zipper), LEA and AP2/ERF (Apetala2/ethylene response factor) gene families (Zhuang et al., 2008; Hu et al., 2012; Lan et al., 2013).

In particular, the AP2/ERF superfamily, which belongs to plant-specific TFs, shares a conserved AP2/ERF domain containing 50–60 amino acids (Sakuma et al., 2002). The AP2/ERF superfamily plays a significant role in the transcriptional regulation of multiple biological processes related to various environmental stimuli responses (Sakuma et al., 2002; Mcgrath et al., 2005; Licausi et al., 2013). According to domain feature and gene function, the AP2/ERF superfamily can be divided into two families, AP2 and ERF (Zhuang et al., 2008). The AP2 family includes: (1) AP2 subfamily containing two repeated AP2/ERF domains are mainly involved in plant developmental processes; (2) RAV subfamily contain a single AP2/ERF domain and a B3 domain that is conserved in other plant-specific TFs, which are mainly involved in plant physiological and developmental pathways. The ERF family harboring a single AP2/ERF domain consists of DREB, ERF subfamily and soloist genes. DREB are mainly involved in abiotic stress responses such as cold and osmotic stresses. ERFs are mainly involved in plant responses to biotic and abiotic stresses, such as pathogen, salt, and drought stresses (Toshitsugu et al., 2006; Licausi et al., 2013; Thirugnanasambantham et al., 2015). ERF subfamily has been a research hotspot and extensively applied to plant genetic engineering/breeding for years, as they act as an important switch in activating or repressing the expression of target genes by specifically binding to *cis*-acting elements (Sakuma et al., 2002; Mcgrath et al., 2005; Licausi et al., 2013). However, downstream genes and pathways, which are regulated by the ERF gene family under abiotic stresses, are litter known in poplar.

*ERF76* is an important member of poplar ERF subfamily. In our previous study, we have validated that the *ERF76* gene responses to salinity stress in leaf, stem and root tissues (Yao et al., 2016b). Under salt stress, the transgenic poplar and tobacco over-expressing *ERF76* gene are superior in plant height, root length and fresh weight than those of non-transgenic plants (Yao et al., 2016a,b). A total of 375 DEGs were identified from transgenic poplar by RNA-seq (Yao et al., 2016b). In the present study, we focused on three aspects: (1) Identification of DEGs in leaves of di-haploid *Populus simonii × P. nigra* under respective stress of NaCl, KCl, CdCl_2,_ and PEG; (2) Examination on the expression pattern of the ERF gene family under the stresses; (3) Validation of the connection between the ERF gene family and the DEGs.

## Materials and Methods

### Plant Materials

The hydroponic poplar cuttings from identical WT poplar and transgenic poplar were cultured for multiple stresses treatments. The twigs of di-haploid *P. simonii × P. nigra* were cultured with water to regenerate new leaves and roots in the greenhouse with 60–70% relative humidity, 16/8-h light/dark cycles, and an average temperature of 25°C for 1 month. The WT poplar seedlings were divided into five groups of 10 plants, and subjected to the following treatments: 150 mM NaCl, 150 mM KCl, 150 μM CdCl_2_, 20% PEG6000 and water (control) for 24 h. Transgenic poplar seedlings were divided into six groups of 10 plants and treated with 150 mM NaCl and water as mentioned above with three biological repeats each. After treatment, leaves were harvested from 10 plants of each group, and shipped to GENEWIZ Company^1^ for RNA sequencing by Illumina HiSeq2500 technology.

### Gene Expression Analysis Using RNA-Seq

The expression of stress-related genes was profiled by RNA-Seq. RNA library construction was conducted as described by Yao et al. (2016b). The mRNA abundances were quantified as fragment per kilo bases per million reads (FPKM). The DESeq algorithm in Bioconductor^2^ was applied for RNA-Seq data analysis. DEGs were identified by comparison between control and each treatment. We used two stringent standards for DEG selection: false discovery rate (FDR) ≤ 0.05 and fold change (in terms of log ratio of gene expression) ≥ 2. Under each treatment corresponding DEGs were listed in **Supplementary Data Sheet S1**.

To facilitate understanding of gene expression patterns, we conducted hierarchical clustering of the DEGs across the four stresses and control using Gene Cluster 3.0 software. We also performed gene set enrichment analysis using gene ontology (GO) database^3^. Significant pathway enrichment analysis was conducted using kyoto encyclopedia of genes and genomes (KEGG) database^4^. And corrected *p*-value of significantly enriched GO terms and pathways was controlled at 0.05.

In addition, we examined expression pattern of the ERF gene family in response to the four stresses. Theoretically, if there is at least one molecular (or one copy) in a molecular population, we have a chance to detect it. In fact, RNA-Seq is randomly sampling process where molecules with high frequencies have higher chances to be sampled for sequencing. We focused on the ERFs whose FPKM ≥ 4 in at least one stress, applying the fold changes of FPKM to claim URG and DRG expression (Jiang et al., 2011).

Venn diagrams of DEGs and ERF gene family under the four stresses were drawn using Venn Diagram Generator^5^. Boxplot graphs of the ERFs and the DEGs under the four stresses were generated with boxplot function in R software.

### Morphological, Physiological and Biochemical Characterization

Since *ERF76* gene is an important member of the ERF gene family and we have successfully cloned and transferred the gene into poplar (Yao et al., 2016b), we thus focused on validation of the function of *ERF76* gene.

We compared morphological traits between transgenic poplar and WT plants. The hydroponic poplar cuttings from identical WT poplar and transgenic poplar divided into six groups of 10 plants. One group is from wild type poplar twigs and five groups are from independent transgenic poplar twigs. After 1 month, the root length and root number of TLs were measured, compared to WT. We also collected the fully expanded leaves from same plant position (at apical fifth position) for stomata observation. The protocols of stomata observation were as follows: (1) the leaves were first fixed in 25% glutaraldehyde for 24 h and then dehydrated in 30, 50, 70, 85, 95, 100%, and 100% ethanol for 15 min each, respectively; (2) the leaves were treated with isoamyl acetate: ethanol (1:1) mixture and 100% isoamyl acetate for respective 15 min; (3) the long axis and short axis of stomata distributed in randomly selected fields were measured by a scanning electron microscope with 40× objective (Olympus BX43 LM with a DP26CU camera; Tokyo, Japan). The size of stomata was calculated by structuring an ellipse being similar with targets’ inscribes ellipse with long axis and short axis, and stomatal frequency was counted on the same leaf surface at a constant position within leaves.

We further characterized the transgenic poplar overexpressing *ERF76* gene in terms of physiological and biochemical traits, compared to WT plants. The physiological and biochemical traits were determined according to the description by Yao et al. (2016a).

### RT-qPCR

To examine the reliability of RNA-Seq data, we profiled the poplar ERF gene family by RT-qPCR in response to NaCl stress. Moreover, in order to further validate the connection between the ERFs and the DEGs, we quantified gene expression of six stress-related DEGs and a few reprehensive stress-related genes in transgenic poplar by RT-qPCR. Actin (ACT) and elongation factor 1 (EF1) are used as internal control (Regier and Frey, 2010). The primer sequences are listed in **Supplementary Table S1**. The RT-qPCR was performed according to our previous experimental procedures (Yao et al., 2016b).

## Results

### Expression Patterns of DEGs in Multiple Stresses

**FIGURE 1:**
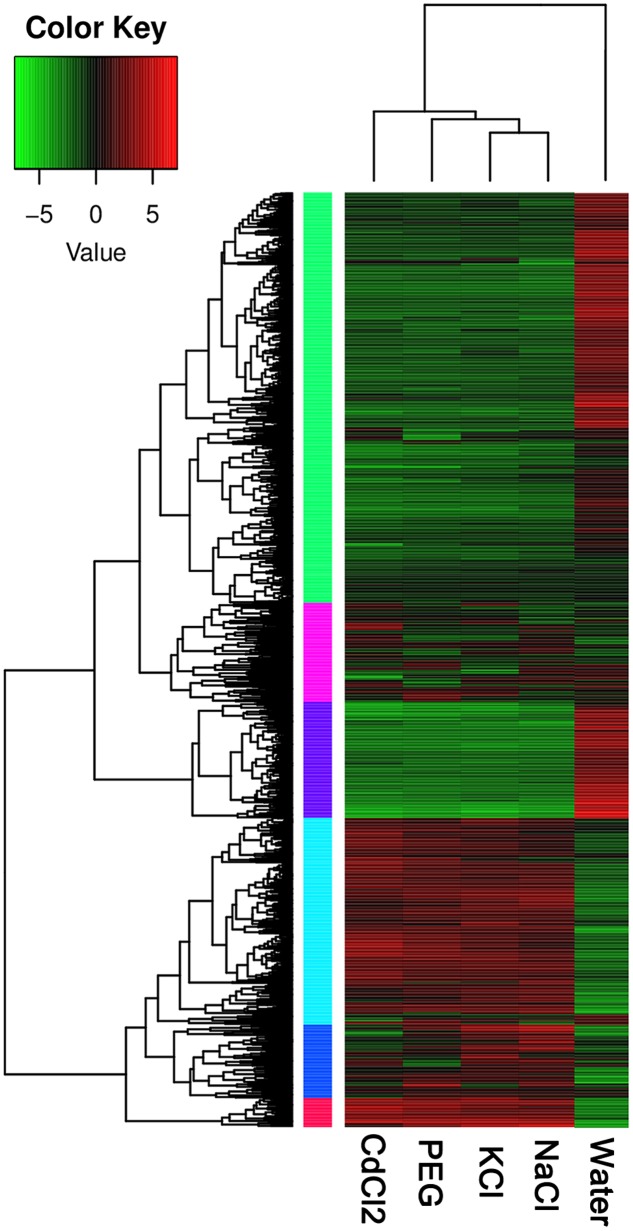
**Heatmap of DEGs under the four stresses in *Populus simonii × P. nigra*.** The expression levels are quantified as log2 FPKM based on RNA-Seq. Red and green colors indicate over- and low-expression, respectively. The colorful vertical bars denoted different gene clusters.

In order to identify gene expression patterns of the DEGs across the four stresses in *P. simonii × P. nigra*, we applied cluster analysis. The results from RNA-Seq gave rise to a profile of 65535 polar genes. We identified 939 DEGs by at least one stress. Based on the expression level, the DEGs can be classified into six clusters in the heatmap (**Figure 1**). The cluster 1 and 3 are lowly expressed in the four stresses; in contrast, cluster 4 and 6 are over-expressed in response to stresses. The other two clusters fall in between the two extremes. In addition, NaCl and KCl stress are clustered together, indicating that the DEGs share similar mechanisms upon the two stresses. DEGs with CdCl_2_ and PEG stress belong to different clusters (**Figure 1**).

Results from fold changes indicated that more DRGs are detected than URGs. There are 404, 557, 653, 584 significantly DEGs detected by NaCl, KCl, CdCl_2_, PEG stress, respectively (**Supplementary Data Sheet S1**). Under NaCl stress, 159 DEGs were up-regulated and 245 DEGs were down-regulated. Under KCl stress, 228 genes were up-regulated and 329 were down-regulated. Upon CdCl_2_ stress, there were 234 URGs and 419 DRGs. By PEG stress, 203 URGs and 381 DRGs were identified (**Supplementary Table S2**). The numbers of DRG are 1.54, 1.44, 1.79, 1.87 times higher than those of URG by NaCl, KCl, CdCl_2_, and PEG stress, respectively.

**FIGURE 2:**
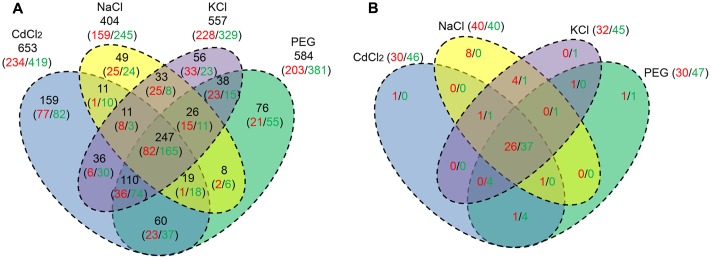
**Venn diagram of the URG and DRG under the four stresses.** The numbers in red and green indicate URG and DRG, respectively. **(A)** A total of 939 the DEGs shared by at least one stress. The numbers in black denote respective the sum of URG and DRG; **(B)** A total of 81 ERFs shared by at least one stress.

Regarding the difference of DEGs in response to the stresses, the results are summarized in **Figure 2A**. A total of 247 DEGs (26.3%) including 82 URGs and 165 DRGs are shared by the four stresses (**Supplementary Data Sheet S2**). We found 11 (8 URGs/3 DRGs), 110 (36/74), 19 (1/18), 26 (15/11) DEGs shared by three out of four stresses, respectively. The numbers of DEGs shared by two out of four stresses are 11 (1/10), 36 (6/30), 60 (23/37), 8 (2/6), 38 (23/15), 33 (25/8), respectively. In addition, as many as 49 (25/24), 56 (33/23), 159 (77/82), 76 (21/55) DEGs are specific to NaCl, KCl, CdCl_2_, and PEG stress, respectively (**Figure 2A**).

In order to understand gene functions, we performed gene set enrichment analysis. We have identified enriched functional categories based on GO terms for each stress (**Supplementary Table S2**). Regarding the significant (corrected *P*-value < 0.05) GO terms shared by the four stresses, the largest fractions (72.9–80.8%) of the GO terms are associated with molecular functions related to abiotic stresses tolerance, such as catalytic activity, oxidoreductase activity, transporter activity, POD activity, oxidoreductase activity, transferase activity, and hydrolase activity. The second largest fractions (17.6–26.4%) are pertinent to biological processes, such as defense response, response to stimulus, response to stress, single-organism process, localization, transport etc. The least percentages (0.005–0.02%) are involved in cellular components including membrane (**Supplementary Table S2**).

We further investigated pathways significantly enriched based on the DEGs. The results indicated that there were 11 (42 DEGs), 20 (57), 20 (73), 16 (53) such pathways by NaCl, KCl, CdCl_2_, PEG stress, respectively (**Supplementary Data Sheet S3**). Under NaCl stress, the DEGs are mainly involved in pathways such as phenylpropanoid biosynthesis (13 DEGs, 30.95%), phenylalanine metabolism (10 DEGs, 23.81%) and starch and sucrose metabolism (five DEGs, 11.90%). Under KCl stress, the results are similar to those by NaCl stress, except for the emergence of plant hormone signal transduction (seven DEGs, 12.28%). Under CdCl_2_ and PEG stresses, the enriched pathways are similar to those of the KCl stress, although the percentages of DEGs vary (**Supplementary Table S2**).

### Expression Pattern of ERFs under Abiotic Stresses

We profiled the ERF gene family by RNA-Seq under the four stresses, compared to the control. Among 175 ERFs, 21 genes display no expression and 81 ERFs whose FPKM ≥ 4 are expressed in at least one stress across the four stresses (**Supplementary Data Sheet S4**). Out of the 81 ERFs, as many as 40, 32, 30, 30 genes were found to be up-regulated in *P. simonii × P. nigra* by NaCl, KCl, CdCl_2_, and PEG stress, respectively. And the numbers of downed-regulated ERFs were 1.0, 1.41, 1.53, 1.57 times greater than those of up-regulated ERFs under the four stresses, respectively, which shares similar trend with the down/up ratio of DEGs (**Supplementary Table S3**). A total of 63 (26/37) URG/DRG was found in common across the four stresses. There are corresponding 1 (0/1), 1 (1/0), 4 (0/4), 2 (1/1) genes showed the same expression pattern across all combinations of three stresses. The numbers of corresponding genes shared by all combinations of two stresses were 5 (4/1), 1 (1/0), 0 (0/0), 5 (1/4), 0 (0/0), 0 (0/0), respectively. There are 8 (8/0), 1 (0/1), 1 (1/0), 2 (1/1) ERFs that are specific to NaCl, KCl, CdCl_2_, and PEG stress, respectively (**Figure 2B**). In addition, the up- and down-regulated ERFs in four FC regions were counted. There exist 15, 10, 9, 10 up-regulated ERFs whose log2 ratio is higher than 2 with the four stresses, respectively (**Supplementary Table S3**). A total of eight up-regulated ERFs are shared by the four stresses (**Supplementary Table S4**). And there are 20, 20, 23, 22 down-regulated ERFs whose |log2| is higher than 2 found under the four stresses, respectively (**Supplementary Table S3**). As many as 16 down-regulated ERFs are shared by the four stresses (**Supplementary Table S4**).

We also conducted RT-qPCR to examine the relative expression levels of 175 poplar ERF genes under NaCl stress. There are 73 genes in response to salt stress, including 33 URGs and 40 DRGs (**Supplementary Figure S1**). Among the 73 ERFs, the expression pattern of 68 ERFs detected by RT-qPCR is in accord with the results of RNA-Seq, which ensures the reliability of RNA-Seq data.

### The Relation of ERFs and DEGs

**FIGURE 3:**
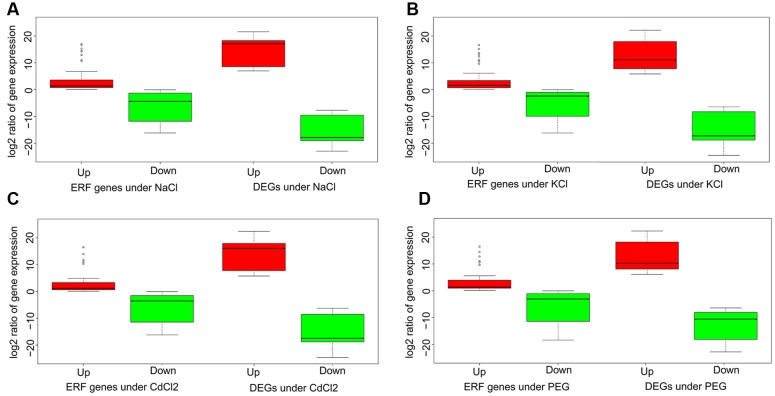
**Boxplots of the ERFs and the DEGs under the four stresses.** The *y*-axis is log ratio of gene expression under stress to that in the control. **(A–D)** denote NaCl, KCl, CdCl2, and PEG stress, respectively. Red and green colors indicate URG and DRG, respectively.

Understanding the connection between the ERFs and DEGs is critical to biology functions related to abiotic stresses tolerance. Consider relative expression level as fold change between a stress and the control in terms of log2 ratio of FPKM, we compared the ERFs and DEGs by respective up- and down-regulated gene groups. The results indicated that there exists a consistent pattern for the up- and down-regulated genes in both ERFs and DEGs across the four stresses (**Figure 3**). In general, the differential expression between the up- and down-regulated genes in ERFs is significantly smaller than that in DEGs. The relative expression levels of up-regulated DEGs are 3.4-fold higher than those of ERFs. Conversely, the relative expression levels of down-regulated DEGs are 2.0-fold lower than those of ERFs. For the ERFs, the variation of up-regulated gene expression is remarkably smaller, compared to down-regulated groups (**Figure 3**). The results clearly indicate the correlations between the ERFs and DEGs expression.

### Morphological, Physiological and Biochemical Analysis of Transgenic Poplar

**FIGURE 4:**
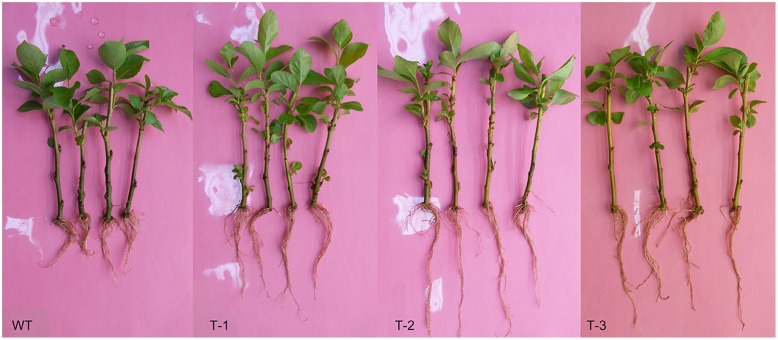
**Morphological traits of transgenic poplar and WT.** T-1:3, transgenic poplar overexpressing *ERF76* gene; WT, wild type poplar. It is clear that the roots of transgenic poplar are significantly longer than those of WT.

**FIGURE 5:**
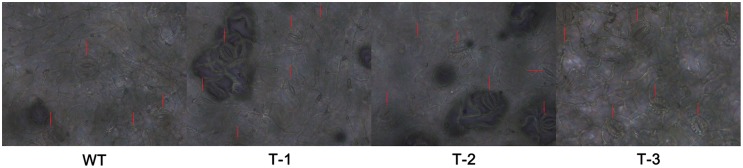
**Comparison of stomatas between transgenic poplar and WT.** T-1:3, transgenic poplar overexpressing *ERF76* gene; WT, wild type poplar. The length, width, size, and density of stomata in TL are markedly higher than those in WT.

We conducted morphological measurements of hydroponic transgenic poplar (TL) and WT poplar. The results indicate the root lengths of TL are 1.29–1.51 times higher than those of WT, although there is no significant difference in root numbers between TL and WT (**Figure 4**, **Supplementary Table S5**). The stomata measurements in leaves indicate that long axis, short axis and size of stomata in TL leaves are respective 1.22–1.37, 1.07–1.26 and 1.30–1.60 times higher than those in WT (**Figure 5**, **Supplementary Table S5**). In addition, stomata frequency in the same area of leaves from TL is 1.25–1.50 higher than that from WT (**Figure 5**). Considerable morphological variations indicate that transgenic poplar is superior to wild type poplar in morphological traits.

**FIGURE 6:**
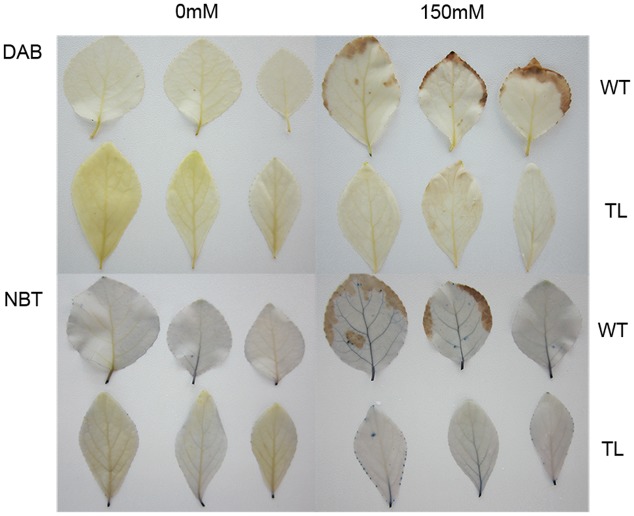
**Histochemical staining of poplar leaves.** The genetically identical poplar seedlings were treated with water (control) and 150 mM NaCl for 24 h, followed by the histochemical staining of DAB and NBT, respectively. TL, transgenic poplar overexpressing *ERF76* gene; WT, wild type poplar. The staining of WT is deeper than that of TL under salt stress condition.

We further investigated the physiological and biochemical traits of TL compared to WT. TL displayed better physiology states, which showed higher POD activity, SOD activity, proline content, and RWC as well as lower MDA content and relative electronic conductivity under salt stress, than those of WT (**Supplementary Figure S2**). As for histochemical staining, the leaves from WT were stained darker in both DAB and NBT staining than those of TL under salt stress condition. However, there was no difference in staining degree between TL and WT under normal condition (**Figure 6**). These results indicated that transgenic poplar is superior to WT in physiological and biochemical traits.

### Gene Expression Analysis of Transgenic Poplar

**FIGURE 7:**
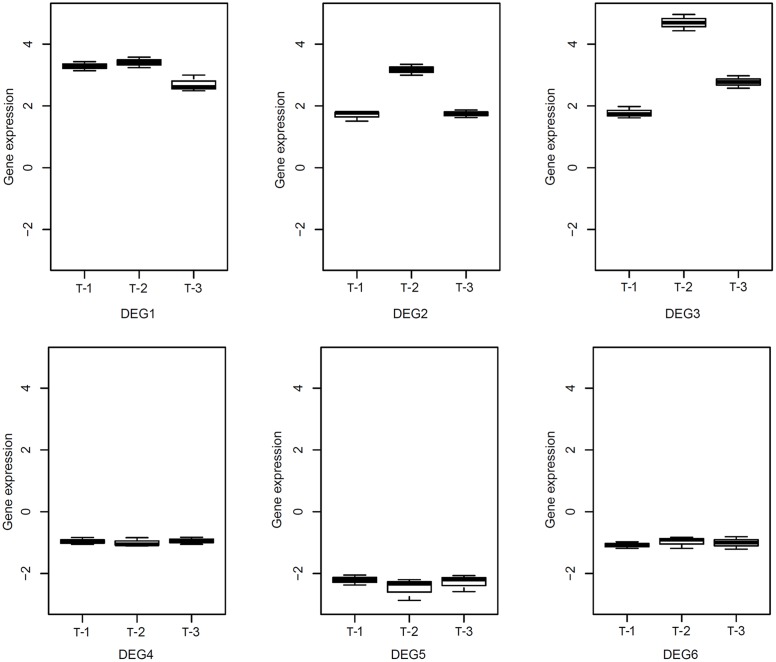
**Relative expression level of reprehensive DEGs in transgenic poplar detected by RT-qPCR.** T-1:3, transgenic poplar overexpressing *ERF76* gene.

We conducted RNA-Seq to check the expression changes of 247 DEGs in TLs with salt stress, which are shared by the four stresses. Based on FPKM ≥ 4, a total of 108 genes including 54 URGs and 54 DRGs were founded in TLs (**Supplementary Data Sheet S2**), and 88 of the 108 DEGs share same expression pattern in TL and WT under salt stress. As many as 38 genes are significantly differentially expressed in both WT and TL by salt stress. Moreover, we determined relative expression levels of six DEGs including three URGs and three DRGs in transgenic poplar by RT-qPCR. The results indicate that the relative expression levels of the three URGs in TLs are 1.42–4.96 times higher than those in WT, and the relative expression levels of three DRGs in TLs are 0.81–2.87 times lower than those in WT (**Figure 7**), which are in accord with the results of RNA-Seq.

To examine the regulatory functions of *ERF76* gene, we determined relative expression levels of *ERF76* gene and several stress-related genes in TL by RT-qPCR. Results indicate that the relative expression level of *ERF76* gene in TL is 9.23–11.14 times higher than that in WT. And the relative expression levels of stress-related genes including POD-related genes, SOD-related genes, HRG-related genes, GST-related genes, LEA-related genes, P450-related genes in TL are 1.26–5.46, 1.21–7.95, 1.18–1.79, 1.40–3.39, 1.53–2.5 and 1.68–2.21 times higher than those in WT, respectively (**Supplementary Figure S3**). These results indicate that *ERF76* gene plays an important role in regulating the stress-related genes.

## Discussion

In this study, we systematically scrutinized DEGs across the four abiotic stresses, followed by examination of ERF gene family expression. Among 939 DEGs, as many as 247 DEGs (26.3%) are shared by the four stresses. These DEGs are mostly involved in molecular functions including catalytic activity, oxidoreductase activity, transporter activity, POD activity, oxidoreductase activity, transferase activity, and hydrolase activity, followed by biological processes such as defense response, response to stimulus, response to stress, single-organism process etc. These biological responses could improve stress tolerance in plants by adjusting the cellular redox state, preventing damage to host cellular components, and protecting osmotic adjustment of plant cells (Jiang and Deyholos, 2006; Shinozaki and Yamaguchi-Shinozaki, 2007; Boscaiu et al., 2008; Golldack et al., 2014).

Transcription factors have been identified as important switches in activating or repressing the expression of stress-related genes in the biotic and abiotic stress responses in plants for years (Singh et al., 2002; Dietz et al., 2010). In the present study, eight ERF genes from poplar were significantly (|log2Ratio|> 2) URGs shared by NaCl, KCl, CdCl_2_, and PEG stress. Among these URGs, *ERF24* gene (Potri.004G051700.1) is the best BLAST hit in *AtERF2* gene (AT5G47220.1) from *Arabidopsis thaliana*, which belongs to ERF B-3 and functions as activator of GCC box-dependent transcription in *Arabidopsis* leaves (Fujimoto et al., 2000). The gene (Potri.018G038100.1) is the best hit in *ERF016* gene (AT5G21960.1) from *A. thaliana*, which belongs to DREB A-5 and binds to the GCC-box pathogenesis-related promoter element (Bae et al., 2003; Nakano et al., 2006). *ERF31* gene (Potri.002G039100.1) and the gene (Potri.011G061700.1) shared high identify with *AtERF1* gene (AT3G23240.1) from *A. thaliana*, which belongs to ERF B-3, acts as activator of GCC box-dependent transcription (Fujimoto et al., 2000) and integrates signals from ethylene and jasmonate pathways in plant defense in *Arabidopsis* (Lorenzo et al., 2003); *DREB14* gene (Potri.003G139300.1) is the best hit in AP2/ERF gene (AT1G64380) from *A. thaliana*, which belongs to DREB A-6, and is up-regulated in response to a plant-defense elicitor chitin (Nakano et al., 2006; Libault et al., 2007). *ERF76* gene (Potri.005G195000.1) is significantly induced by salt stress (Yao et al., 2016b), which shared high identify with *ERF110* gene (AT5G50080.1) from *A. thaliana*, probably acts as a transcriptional activator, and may be involved in the regulation of gene expression by stress factors and by components of stress signal transduction pathways (Nakano et al., 2006; Li et al., 2012). Characterization and function analysis of significantly changed ERF gene by abiotic stress will help understand gene regulatory mechanism in stress responses.

Stomata are the microscopic pores sandwiched between a pair of guard cells and spaced by a minimum of one epidermal pavement cells on the surface of leaves (Nadeau and Sack, 2003; Casson and Gray, 2007). Stomata formation and differentiation occurred during organ development by a series of flexible asymmetric cell divisions and progressive fate acquisition events (Nadeau, 2008). The variation of stomatal size and density on the leaf surface play a fundamental role in the regulation of transpiration and photosynthesis rate as well as the control of plant water balance in response to environmental stimuli (Casson and Gray, 2007; Nadeau, 2008; Pillitteri and Torii, 2012; Rajabpoor et al., 2014). The transgenic poplar overexpressing *ERF76* gene held higher stomatal abundance and size in the leaves than those of WT under both normal and salt stress conditions, which means transgenic poplar held better regulative capability in trade-off between water loss and carbon acquisition than WT.

Plants challenged to various environment stresses can induce a series of physical, chemical, and biological processes (Boscaiu et al., 2008; Rajabpoor et al., 2014). These stress response mechanisms and complex multicomponent signaling pathways can produce many cations and anions restoring ion homeostasis, some osmolytes adjusting intracellular osmotic balance, and several ROS scavenger controlling cellular redox states *in vivo*, as well as the control of water loss by stomatal closure, raised stomatal resistance, leaf area changes etc (Boscaiu et al., 2008; Golldack et al., 2014; Rajabpoor et al., 2014). POD, SOD and GST are typical ROS-scavenging enzymes with functions in detoxification of ROS, which can prevent severe oxidative damage to host cells (Blokhina et al., 2003; Jiang and Deyholos, 2006; Golldack et al., 2014). MDA is the principal product of polyunsaturated fatty acid peroxidation, which acts as toxic molecule and biological marker of oxidative stress (Rio et al., 2005). After salt stress, higher amounts of antioxidative enzymes including POD, SOD, GST as well as lower MDA content were produced in salt tolerant variety (S1) than those in salt susceptible variety (ATP) under NaCl salinity (Sudhakar et al., 2001). Transgenic poplar overexpressing *ERF76* gene displayed better physiology states including higher POD activity, SOD activity, proline content, and RWC as well as lower MDA content, relative electronic conductivity under salt stress condition, compared to WT. In addition, the histochemical staining results indicated ROS accumulation in transgenic poplar was lower than that in WT. Those indicated transgenic poplar overexpressing *ERF76* gene held higher salt tolerance than WT in physiological and biochemical traits.

Many stress-related genes such as HRG, LEA and P450-related genes were involved in improving stress tolerance of plants by increasing the content of osmolytes or protecting macromolecules which regulate cellular homeostasis under stress condition (Craven et al., 2007; Shinozaki and Yamaguchi-Shinozaki, 2007; Rajagopal et al., 2008; Faezah et al., 2012). The relative expression levels of stress-related genes including POD, SOD, HRG, GST, LEA and P450-related genes in transgenic poplar overexpressing *ERF76* gene were proved to be higher than those in WT by both RNA-Seq and RT-qPCR, which demonstrated that the *ERF76* gene plays a significant role in the stress resistance at molecular level.

In summary, a total of 247 stress-related DEGs from di-haploid *P. simonii × P. nigra* were identified across NaCl, KCl, CdCl_2,_ and PEG stress in the present study. Among 175 ERFs, as many as 63 genes were examined to be induced by the four stresses using RNA-Seq. The ERFs and DEGs share similar expression pattern across the four stresses, and the *ERF76* gene plays a significant role in improving salt stress resistance of transgenic poplar. This study explored DEGs by multiple abiotic stresses and clarified the connection between the DEGs and ERFs under abiotic stresses, which gives a rise in understanding the stress response mechanisms in poplar.

## Author Contributions

TJ and BZ designed research. WY conducted experiments and data analysis, and wrote the manuscript. XZ and KZ conducted experiments and data analysis. RL revised the manuscript. All authors read and approved the manuscript.

## Conflict of Interest Statement

The authors declare that the research was conducted in the absence of any commercial or financial relationships that could be construed as a potential conflict of interest.

## Footnotes

1 www.genewiz.com

2 http://www.bioconductor.org/

3 http://geneontology.org/

4 http://www.kegg.jp/

5 http://www.pangloss.com/seidel/Protocols/venn.cgi

## Supplementary Material

**FIGURE S1 | Relative expression levels of ERF gene family in poplar leaves with salt stress by RT-qPCR.**

**FIGURE S2 | Comparisons of physiological traits between transgenic poplar and wild type poplar.** WT, wild type poplar; T1-5, transgenic poplar overexpressing *ERF76* gene.

**FIGURE S3 | Relative expression levels of stress-related genes in TLs by RT-qPCR.** T1-3, transgenic poplar overexpressing *ERF76* gene.

**TABLE S1 | Primer sequences of genes.**

**TABLE S2 | DEGs, significantly (Corrected *P*-value ≤ 0.05) enriched GO terms and pathways under the four stresses.**

**TABLE S3 | The number of up/down-regulated ERF genes in different log ratio regions under the four stresses.**

**TABLE S4 | ERFs (|log2Ratio| >2) shared by the four stresses.**

**TABLE S5 | Morphological variation between transgenic poplar and wild type poplar.**

**DATA SHEET S1 | The corresponding DEGs under each stress.**

**DATA SHEET S2 | The 247 DEGs shared by the four stresses and 108 out of the 247 DEGs detected in TL by salt stress.**

**DATA SHEET S3 | The corresponding pathways under each stress.**

**DATA SHEET S4 | The poplar ERFs response to the four stresses.**

## Abbreviations

DAB: diaminobenzidine
DEG: differentially expressed gene
DRG: down-regulated genes
ERF: ethylene response factor
GST: glutathione S-transferase
HRG: hydroxyproline-rich glycoprotein
LEA: late embryogenesis abundant protein
MDA: malondialdehyde
NBT: nitrotetrazolium blue chloride
P450: cytochrome P450 protein
POD: peroxidase
REC: relative electrical conductivity
ROS: reactive oxygen species
RWC: relative water content
SOD: superoxide dismutase
TF: transcription factor
TL: transgenic poplar overexpressing *ERF76*
URG: up-regulated genes
WT: wild type poplar
