# Supplementary figures and images for: Expression Pattern of ERF Gene Family under Multiple Abiotic Stresses in Populus simonii × P. nigra
Source: Front Plant Sci. 2017 Feb 20;8:181. doi: 10.3389/fpls.2017.00181 (PMC5316532; doi:10.3389/fpls.2017.00181)

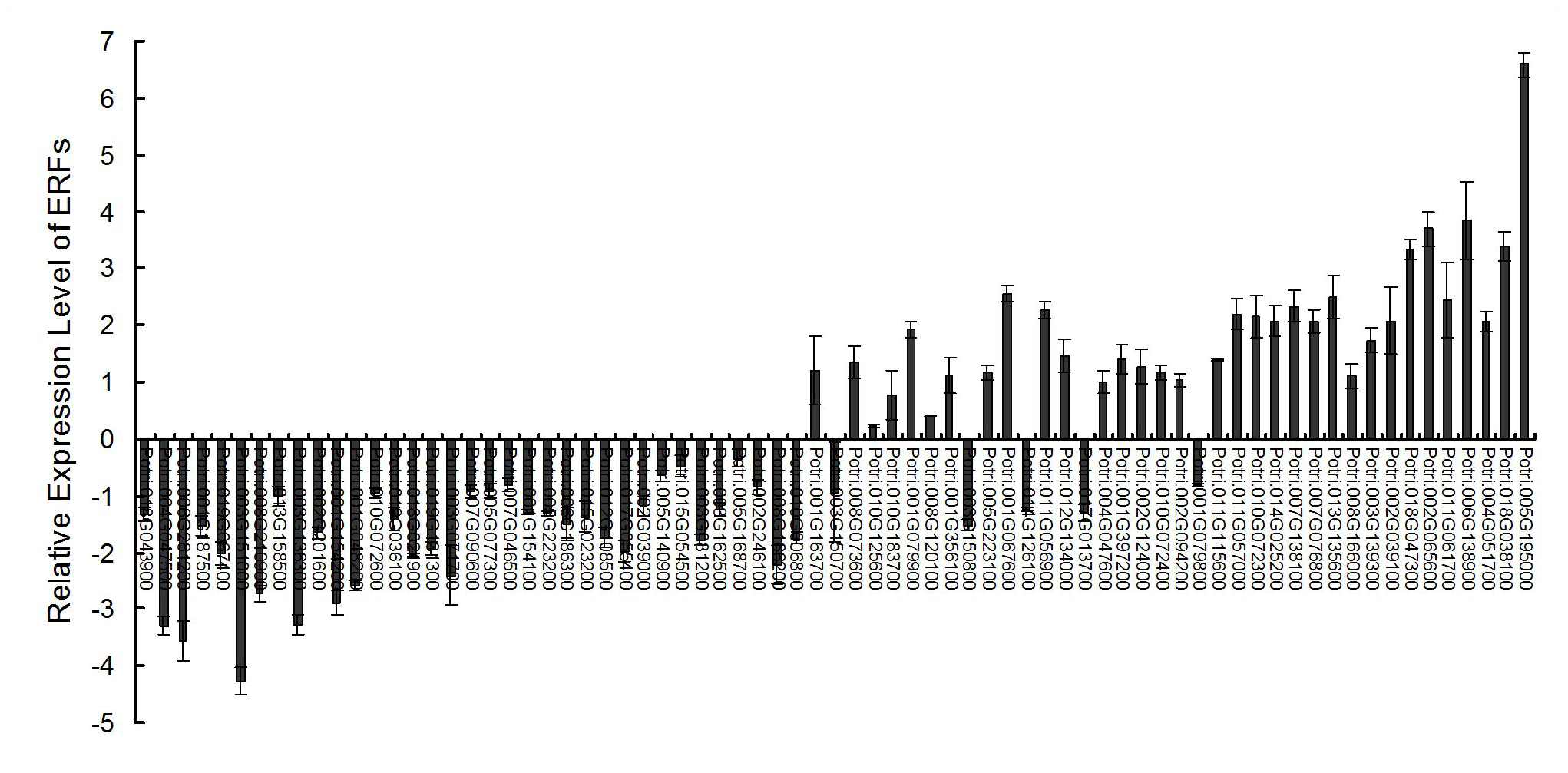

Supplement: Supplementary file 1 [file Image_1.tiff]

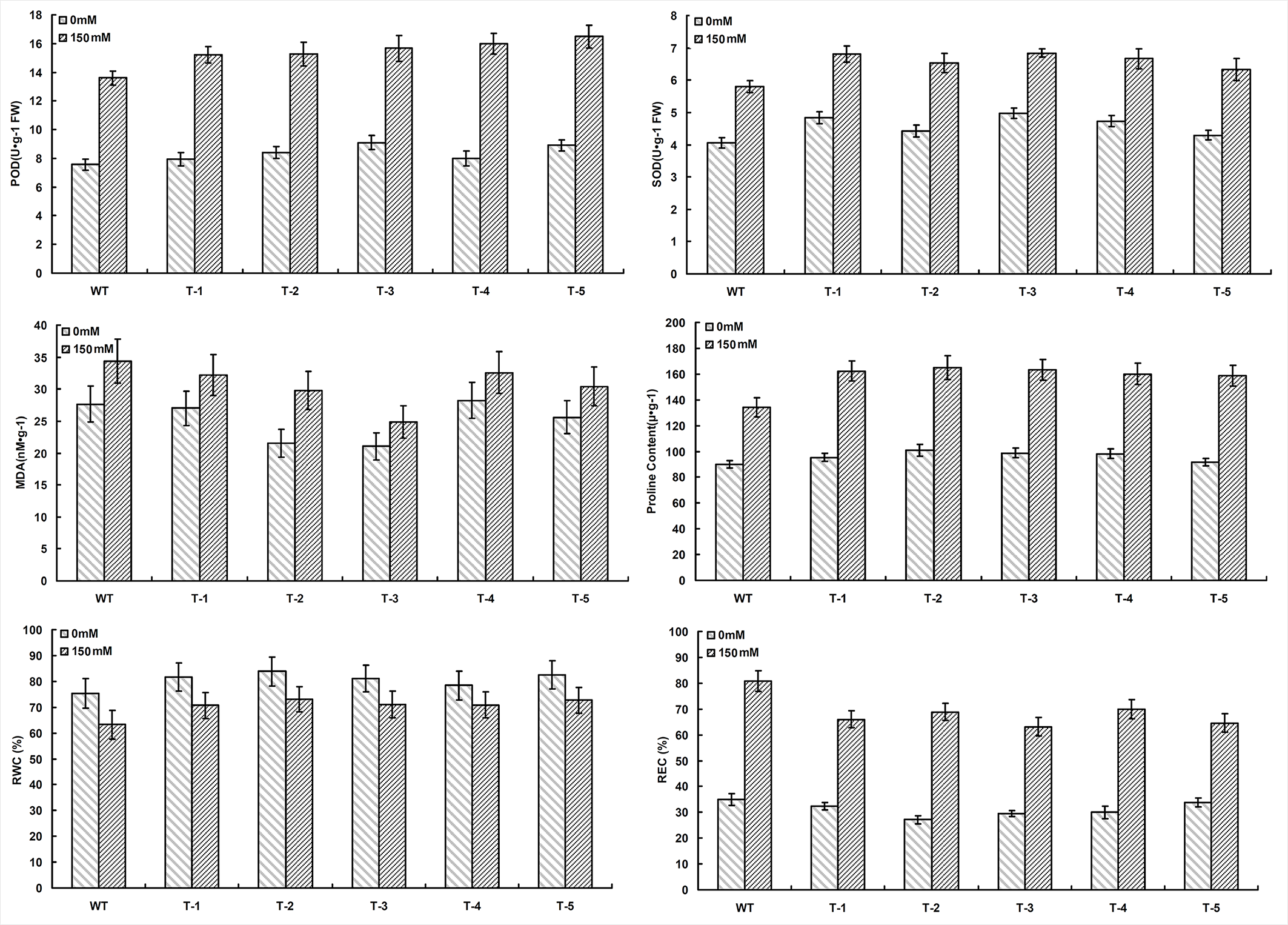

Supplement: Supplementary file 2 [file Image_2.tif]

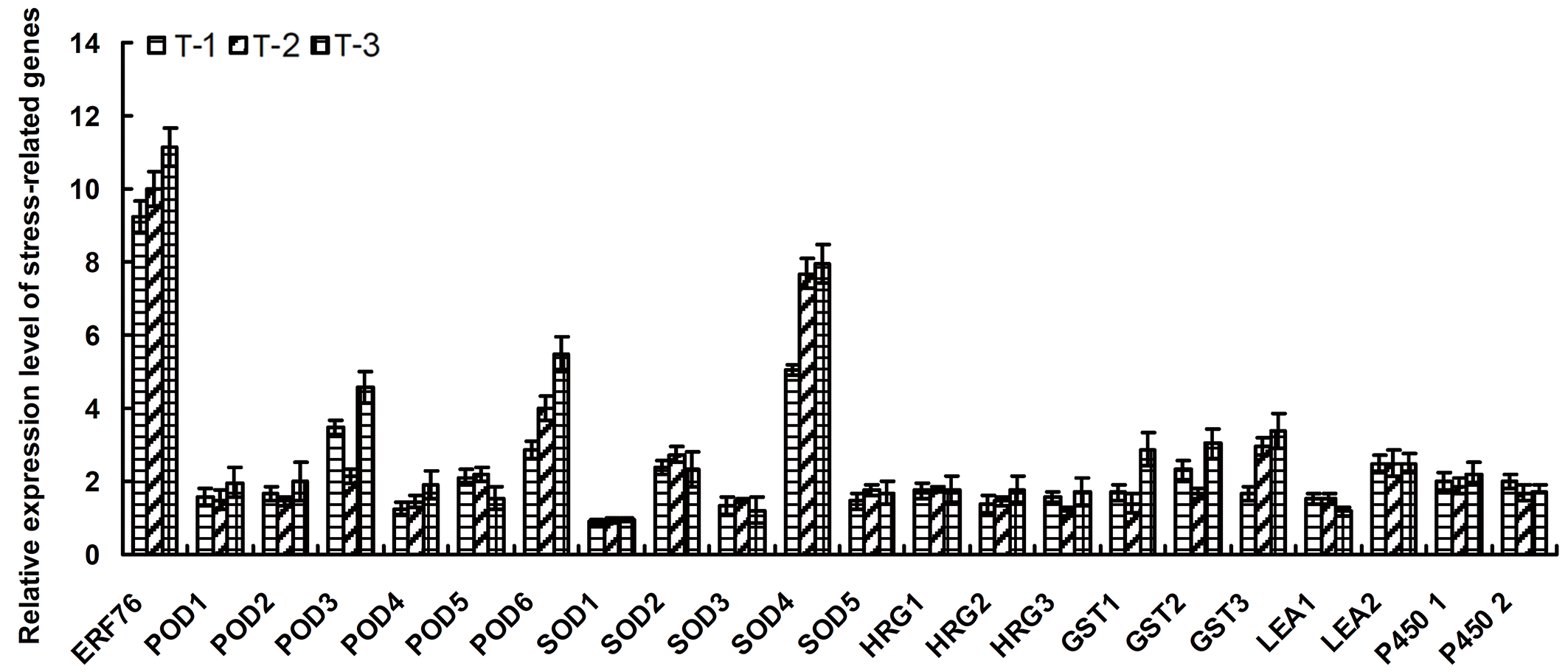

Supplement: Supplementary file 3 [file Image_3.tif]
