# Supplementary material for: Expression Pattern of ERF Gene Family under Multiple Abiotic Stresses in Populus simonii × P. nigra
Source: Front Plant Sci. 2017 Feb 20;8:181. doi: 10.3389/fpls.2017.00181 (PMC5316532; doi:10.3389/fpls.2017.00181)
Supplement: Supplementary file 4 [file Table_1.doc]

Supplemental Table S1 Primers sequence of related genes

| Name | Gene ID | 5’ primers | 3’ primers |
| --- | --- | --- | --- |
| ERF76 | Potri.005G195000 | ATGTGCGTATTGAAGGTGGCG | CCTCCACATGCCGCTGTATTGG |
| ACT | JM986590 | ACCCTCCAATCCAGACACTG | TTGCTGACCGTATGAGCAAG |
| EF1 | FN356200 | AAGCCATGGGATGATGAGAC | ACTGGAGCCAATTTTGATGC |
| DEG1 | Potri.006G055600 | CGACCACTACTTCACCGAGC | CGTTGAAGTAGTAGTAGAAG |
| DEG2 | Potri.008G101000 | GCTGGCATGTGTCTCTTCG | CACAGCATAGCCGAAACAAG |
| DEG3 | Potri.008G116500 | ATTGTGGATGATGTGAGAGC | GCCAACTTCCACATCATCAG |
| DEG4 | Potri.001G032500 | CCTGTGCAATGAGCAACATG | GTTCCTGTGGTATCAGTCAC |
| DEG5 | Potri.010G211900 | CACCACCGCTACCACTTCTC | CTGTAAGGAGTGGAGCGTTG |
| DEG6 | Potri.011G164800 | GCCTTATCCTTTCATCAACC | CGCCGCTGAGTTGATTGTC |
| POD1 | Potri.016G084800 | GTCTTATGCTGACTTCTACC | AATCCAGAACGCTCCTTGTG |
| POD2 | Potri.015G003500 | GGTTCTGTGCTTCTCGACTC | TGTGCCATCTCTTCTTCCAG |
| POD3 | Potri.007G067200 | GCTGAGTCCATTGTTAGATC | GCCTGCCTCTCAATGGAACG |
| POD4 | Potri.016G132700 | CCTCATCAACCATCACTACC | TAGTATTTGCTGGTGCTGTC |
| POD5 | Potri.013G154400 | ATGCTTTTGCTTGGATTAGTC | CAATCACATCATAACCTCTC |
| POD6 | Potri.007G053400 | GTTCCATCTCTTACTTGTTC | CTTGTTGGTTGCAGTGGAGG |
| SOD1 | Potri.003G118400 | GCCTTGCCTGAGATACTTAC | GCTTCAGTCATAGTCTTCAC |
| SOD2 | Potri.005G089600 | GAGTAAGAGGACAGTAGAGG | ATGTCTCCTCCTCCTGGTTG |
| SOD3 | Potri.009G005100 | CTAATGTTGAAGGCGTCGTC | ACGCATCCATTTGTTGTGTC |
| SOD4 | Potri.013G092600 | GAATCTCACTCCTGTCCAAG | CAACACCAAGTAATGGAACC |
| SOD5 | Potri.006G049100 | CTCTCATTCTCCTCTCCGTG | CCAGGCACACCACCAACTTG |
| GST1 | Potri.001G437400 | TGAGAGTGAGAATAGCATTG | GGTAAGGATCAGAAGGCAAC |
| GST2 | Potri.T035000 | ACAGAGGTAGAATCCCACCG | GTGTATGGTAGGTGGTGAAG |
| GST3 | Potri.T149500 | TCCTCTGTTGCCTGAAGATC | AGGTAATAAGCAAGCCATCC |
| HRG1 | Potri.007G113700 | CGTCATCTCCTCCAGTTGCC | GAATGGAATAGAGGGAATAG |
| HRG2 | Potri.006G204300 | CACCTCCGCAACACTACCAC | GTTGCGGATACTGATGTTGG |
| HRG3 | Potri.016G071600 | ATCTTACGGCAGTTCTCCTC | GCGGTGGTACTGTTGTGTAG |
| LEA1 | Potri.002G203500 | CGAGGCAATCAACGGCAGAG | CGATTTCCTGAGCAACATTC |
| LEA2 | Potri.002G165000 | CAGAGGCTACCATCACCGAC | GGCACCTTCATCGGCACATC |
| P450 1 | Potri.003G066400 | CAACACAACCCACCAACAAG | AGTTCGCCTATGAGATTGAG |
| P450 2 | Potri.019G064200 | GTCTCTCTTCCTTGCCTGCG | CTGTAGAAGTGTAGTCATGG |
| 1 | Potri.002G039200 | ACATTTGACACAGCAGAGGAGG | GCCTGCTGTGATCATCTGTTTC |
| 2 | Potri.001G154100 | TGTTCCTTTACGGTGTCCTCAG | TCTCGAATGTCCCCAACCAAAC |
| 3 | Potri.018G085700 | GCCCAGAAAGAAGAATCGGATC | TTCATCGTCGCAGACTTTGGTG |
| 4 | Potri.019G073300 | TCTTATTCTACACCCGAGGCAG | TCGCAACATTCTCAGGTCCAAC |
| 5 | Potri.003G071700 | GCCAAGAGGAAGAGAAAGAACC | GCCTTCGTAAGTCGTTTCTGTG |
| 6 | Potri.002G246100 | AACCAGGAACCATGCCTGATGA | CCCCGATCCTTCTCTTGTGTTT |
| 7 | Potri.006G080300 | GTCCAGTAGATGCCTTGTCCAAG | GACGCACTAGCTGGAACATAACC |
| 8 | Potri.003G121200 | GCCCTAAAAGGTGCTGATGCAGT | TTGGTGGCGAAAGCAGCATTCCT |
| 9 | Potri.011G057000 | ACAAGAATAACAGTTGCGGCGGC | CACCACAAGAAAAACCCATCGGC |
| 10 | Potri.008G071100 | CGAGCTGCCATCATCCTTTATGG | TGGCGTAAATCCTGACGAAGCTG |
| 11 | Potri.003G054400 | GCTCGGATATTGTAAGCTGTGGC | CTATATCCGACACCAATATCGTTC |
| 12 | Potri.002G039300 | GGGGAAATTTGCAGCGGAGATAC | CCTCGTCCAATCCCACTTCTTTC |
| 13 | Potri.005G168700 | CCAGATCCAAACCCAAATCCACC | CAGAGACGGGTTCTATTCTTGGG |
| 14 | Potri.019G075500 | CCAAAGCCAACAAGCTCTAGCTC | GCTTGTTATCCCACTCATCCTCC |
| 15 | Potri.007G138100 | GTTTGGTTGGGCACGTTTGACAC | AGAAACCAGACAGGACCAACAGC |
| 16 | Potri.010G163900 | GAGATTAGAGTGTGGTTGGGGAC | GAGTGCCAGCCTTAAACATTCGG |
| 17 | Potri.004G047600 | GTTTGGCTTGGCACTTTCGACAC | CAGCGGGACCAAGATTAAGATCC |
| 18 | Potri.010G183700 | GCTGCTCTTGCCTATGATGAAGC | GCTTGGCTAGATACATCCTTCCC |
| 19 | Potri.001G069300 | GCACGCACCAACTTTGTCTACTC | CAGCTCCAAATCCTTGAACCCAG |
| 20 | Potri.006G261200 | GCAAGGCTTATGTGTGGACCTAG | CAGTTTGCCTCTGCCTCTTGAAC |
| 21 | Potri.005G223100 | CCGAGATACGTGACCCAACAAAG | CACAGGATTGGCAGTGGAGAATG |
| 22 | Potri.001G067600 | ACGTGACCCTAAAAAGGCAGCTC | GTGTCTGGTGCAAATGATGAGGG |
| 23 | Potri.014G025200 | GAGGGATAAGGATGAGGAAGTGG | CATCTCGCAGCTCATCTTCTTGG |
| 24 | Potri.015G136300 | CTTTCCTATCCAACACCAGCCTC | TTCCTCCTGCCATGTCAACTAGC |
| 25 | Potri.004G051800 | GAAAGTGACCACCAGGCATTACC | GGGAAGTTGAGGTATGTCTTGGG |
| 26 | Potri.013G158500 | GCAGCTCCTTCTTCCACAACTTC | GCGTCGGGTCCTTTTAGTTTCAG |
| 27 | Potri.006G021000 | GTAGCAGTAGCAGTGGAAGTAGC | ACAGCAGCCATTTCAGGGTTAGG |
| 28 | Potri.005G219600 | GGGTCGATTTGCTGCTGAGATTC | CAAAAACACCTGCACCAACCACC |
| 29 | Potri.003G150800 | CTCACCAAAGTCTCCAAGCACTC | TTGTTAGGGTCACGGATCTCAGC |
| 30 | Potri.006G163400 | CTCCTCCTCTTCGCCAACCCCATC | CTGGGCTCCCGGATCTCCGACAC |
| 31 | Potri.008G073600 | GGCTTGGTACTTTTCCTACTGCC | ATCCTCGTACGCACAAACCTCAG |
| 32 | Potri.002G167400 | GTATCGTGGTGTGAGACAAAGGC | CATCTGGGGCATGTTGTTTGCTC |
| 33 | Potri.T050600 | TGCTTATAGAGGGGTTAGGAGGC | CCTTTTCCTACTCTCGGCTTTCC |
| 34 | Potri.013G056700 | GAAGAAACTGCGGAAGCCTCTAC | GGTCCAAAACCCCGTGAAAGATC |
| 35 | Potri.014G047000 | CAATATAGGGGAGTGAGGAG | CAATTCAGGCTCAGGGGATCTTC |
| 36 | Potri.002G065600 | GGCACATTTGATACTGCAGAGGC | GTTGCCTTGGAGATGAGATTGGC |
| 37 | Potri.018G028000 | GATAACAACTCTCCCTTGCCTGC | CTTCTTCCGTACCATTTTCCGGC |
| 38 | Potri.003G079300 | GCTGCAAGACAAGTCAAAAGGGC | TCCTCGTGGTGCCAAAATCCAAC |
| 39 | Potri.006G186300 | GAGGCAGCTATGGCTTATGATAG | CATGGCAAGCACGACTTCAATGC |
| 40 | Potri.017G055400 | GCGGCAGCTTCATTGTTACATCC | GGCTGTGTCTTCGTAGTGCTAAG |
| 41 | Potri.014G046800 | GAGAGAGATGTTGAGGGTGCATC | GGTCATAAGCCAAAGCTGCATCC |
| 42 | Potri.003G077700 | GCGACCATAATAGCAACCTGTCC | TATCAGTTCTGGTGCTGGAGCAG |
| 43 | Potri.006G253800 | GGCAGCAGCAAGAGCATATGATC | GAGCCAGCTTTCTTTTGCCACAC |
| 44 | Potri.019G067400 | CAACTCAGCGTGTCTCAACTTCC | CCGTTTCTATCTCACCCAAACCC |
| 45 | Potri.009G147700 | TTCAAGAGCCCAGAAATGGCAGC | GATTAGGCACCTTCTCTGAGCAG |
| 46 | Potri.013G100300 | CCCTCTAATGTTGCCTGCAAGTG | GATGTCCCCTTCTCTCATTCCAG |
| 47 | Potri.010G046600 | GTTCCGCTAACACTTCCACTCTG | GTGGGGTAGACTAGTGACAGTTG |
| 48 | Potri.006G238600 | GAAACAACCACCACCACAACAGC | CACCACCGCTGAGACAGATATTC |
| 49 | Potri.010G072400 | GACATTTGATACGGCAGAGGAGG | CTTGCTGCCGTTTCTTCTTCTCC |
| 50 | Potri.019G088000 | CATTGGCTAGGCACGTTTGACAC | GAGCGTTATTGCCATCTACGTCC |
| 51 | Potri.003G179900 | CCAACGAACCCCAATCATCATCG | GTTCTTGATGGTCATCGTTCCCC |
| 52 | Potri.010G186400 | GCCTTCATCCTTTATGGCTCCAG | GAGTAAATCCCGATGAAGCTGCC |
| 53 | Potri.007G043400 | GCCTTGGTTGCGTCTATTTCTCC | AAGTTCCCAGCCACAAACGAGAC |
| 54 | Potri.008G210900 | CAAGCTACACCATCCAAGTCCAG | GCTTCATCATATGCTTTGGCGGC |
| 55 | Potri.005G077300 | GCATTGGGGAAAATGGGTTGCTG | CAACAGAGGAATGGAGAGGCTTG |
| 56 | Potri.019G075600 | GTTGCAGCATTGCACCTTAGAGG | TGACAACCCAACCCTAACAGGAC |
| 57 | Potri.003G081200 | GGTCCCATCACTCAAAACCGATC | TTCTTCGCTGGGTCCCTAATCTC |
| 58 | Potri.012G032900 | GACTTACTGCCCAATCAACCACC | GTTAAAGCATCTGGCCCTCGAAG |
| 59 | Potri.003G220200 | CAAAACTCTTCCACAACTCCCCC | GAGGGATGGAACTGAGAAGTGAC |
| 60 | Potri.013G101200 | GAAATGGTGGATAGTTTGCCCCG | CACAGGCTCATCCAACAGAAGAG |
| 61 | Potri.003G054100 | CTGAAATGGAAGATAGCCTCCCC | CATTTTTGGCGAGTCCAGTGGTG |
| 62 | Potri.012G134100 | CTACTGCTGAAATGGCTGCTAGG | TCATCTCCCGTCCTCATCAACTC |
| 63 | Potri.018G043900 | CGCTGCTAAAGCTGCTCAAATGG | CGCCGCCACAACAGTGATTTTCC |
| 64 | Potri.005G223300 | GATCAAGGAAAAGGAAGCCGCTC | TTGAGGACAGCCTTAGAGCCATG |
| 65 | Potri.017G087800 | CTGATTTCCTCGACCATCCAGAC | CTTCTTCTGCTTCACCTCCCAAG |
| 66 | Potri.002G172300 | CGCATTTGGCTAGGCTCATTTCC | GCCACCACCATTGTTTGTTTCGC |
| 67 | Potri.002G039100 | GAACCTTTGATAGTGCAGAGGCG | CCCAAATCCTCCAAAACCACCAC |
| 68 | Potri.002G029400 | GCTGCTCGTAAACTATATGGCCC | TTGATTACGTCTCCACTTCGCCC |
| 69 | Potri.019G067400 | CCGGTAAGAAGACCAGAGTATGG | GACTCGCAATCCTCAAATCCGAC |
| 70 | Potri.004G047500 | CTTCCACTTCTGGTCTCTCTCTG | ACGAAGCCGGAGCAACATTTTCC |
| 71 | Potri.008G186300 | CATGTTGGTGACTTCTCTGCTGG | GCGGTAGAGTAGTGACAGTTGAG |
| 72 | Potri.005G140900 | GGAGAGAATTTTAGCTGCGGTGG | CAGGCACCTTATTCAGGTCAACC |
| 73 | Potri.001G048200 | CCCTTACAATCCCAATGAGCCAC | CCAGTTACCCTCCTGCCATTTTG |
| 74 | Potri.001G315300 | GGCAAGGATCAAAGATGTGAGCC | GTATTTCATCGGCTGTGTGGTCG |
| 75 | Potri.012G108500 | TTATGGCTCCTCTAGCTCGTTCC | CTGCTGTTGAGAAGATATCGGCC |
| 76 | Potri.005G195000 | GTCATCTGGTGCAACTGCAACTG | CCAGACTCTTGCTGCTTTGTGTG |
| 77 | Potri.008G166200 | CTTTTTGGGCTAATCTCGGAGGC | GCCTCTGCACTATCAAATGTGCC |
| 78 | Potri.014G055700 | GTGAGATCAGAGAGCCAAGGAAG | TTGTTTCGGGGAATGAAGCGGCG |
| 79 | Potri.003G162500 | GTTCAAAGGCACCAAGGCTAAGC | TCTGGTGCAAATGATGAAGGGGG |
| 80 | Potri.002G085600 | AGGGCTCTTCAGCCAATCTCAAC | ATCGGACGGTGAGGAAACCATTG |
| 81 | Potri.003G050700 | CGTATTTGGCTCGGAACATTCCC | CTCGGATTCTGCCACATCTTCAG |
| 82 | Potri.002G172600 | ATTCAAGCAGCAGCAGCAAAGG | GTATTCCATCCACCAGAATCCC |
| 83 | Potri.013G101100 | GTTCATAGCCTACCAAAGCCAG | CCCTGACATTTGCATCCACATC |
| 84 | Potri.002G043300 | CGTCGTTGGCTTGGAACTTTTG | GCCATTCTTGACACCTCATCAG |
| 85 | Potri.018G131400 | CAAGGCACCATCACCATCAATG | TCTGCTCTCAATTCTGGCTCTG |
| 86 | Potri.002G141200 | GCAAATGGGTGTCCGAAATCAG | CCACCTTCACCTTCACAGTATC |
| 87 | Potri.001G092400 | GGTTAGGTACGTATGACACAGC | GCTTGAATCTTGGCATCTACGG |
| 88 | Potri.003G161000 | GCTCTTCTTCTCTCAGGATCAG | GAAATAGATGGGCTCAGTAGCG |
| 89 | Potri.001G154200 | CCCGTGGATGATAATTCTGGTG | TCACACCTCCCAGCTTCTAATG |
| 90 | Potri.015G054500 | CGACAGCAAAATGGCATTGGAG | GTTCGCTCTTCCCATAAATCCG |
| 91 | Potri.007G090600 | GGGTTGCTGAGATTAGACTTCC | GAATGGAGAGGCTTGTAGTCAC |
| 92 | Potri.008G215600 | TACTTGGGCTGCATTGTTCCTG | GAGCTCATCAAAGCCAGGAAAG |
| 93 | Potri.010G125600 | CATCTCTGGCCATCTCTCAATC | TGGACAAGATGCCAAAGGATCG |
| 94 | Potri.014G046900 | TTATACGGGAGTGAGGAGAAGG | GCTTATGAGTCACCCTAACAGG |
| 95 | Potri.003G080600 | CTTCTAACCCAAACCCGCAAAC | ATAGGCTTTAGCGGCTTCAAGG |
| 96 | Potri.001G356100 | CAGTTTGAGCAGTACAGTGGAG | TCTCTGTCATCAACCACCGAAG |
| 97 | Potri.001G004700 | CTTTAACACTCATGTCCCCTCC | TCCAATGCAGCAGCCACTCAAG |
| 98 | Potri.003G151000 | AAGTTTGCAGCAGAGATCCGTG | GATCTTCTTCTCTTCCTGCCTG |
| 99 | Potri.011G115600 | CCTCACTCTTAACATTTCCCGC | CATTCACCATCACCGTTCGATC |
| 100 | Potri.016G126100 | GGAGAAATGGATGGAGACCTTG | CGGAAGACAAGCATTCATGGAG |
| 101 | Potri.019G036100 | GCAGCAGCATTAGTAGTTGTGG | ATGCAAACCCATCGAACACAGG |
| 102 | Potri.001G181500 | CCTGAATTGGCTGGTAAGCTAC | TGGATGGCCTGAATTTGGCTTG |
| 103 | Potri.008G091300 | GGAGTAATGTTGATGGCAGTGG | CTAATCATTGGTGGCTCATCCC |
| 104 | Potri.005G176000 | CCTCGCATTACATTCCTGACAC | ACTGATGGGGGTGATGAGATTG |
| 105 | Potri.011G061800 | AGAGAAAGTGACCACCAGACAC | GGAAGTTGAGGTTTGTCTTGGG |
| 106 | Potri.001G453100 | CATCGACAGCCTCTATTTCTCC | AATATGGTTCATCTGCGTGGC |
| 107 | Potri.003G139300 | CACTCCAGCAACTATTTGGCTC | GCTTGAATCTTGGCATCCACAG |
| 108 | Potri.002G153500 | CACAAACAAGGCACAAGCAACC | GCAGCTTCATCATAGGCTCTAG |
| 109 | Potri.003G150700 | GCTGGATTGAATGAAGCTGCTG | GCATTCTATAAGCCGCCCTATC |
| 110 | Potri.006G054500 | GCTTCTAGCTCTTCTTCCTCTG | CTTCTTTCAGTATGCCCAGCTG |
| 111 | Potri.001G079800 | CAAGATAGGCATTACAGGGGTG | ATCGCTTTGCTTCCTCGTAACC |
| 112 | Potri.004G051700 | AAAGAAGCACTACAGAGGCGTG | TGTGATTCAGGTTGCGATGCTG |
| 113 | Potri.005G233300 | AATTGCCAGTTTCCTGCCTCTC | CATTTACTGCCCCCTTTCCATC |
| 114 | Potri.001G187500 | AGAACTTGCTGCCTCATTACCG | CGGTAACTCCACGATTTCACTC |
| 115 | Potri.008G166000 | GAGCAGCTTATGCAATGAGAGG | CTCAAGCATCTCCTCAAGCAAC |
| 116 | Potri.009G101900 | AGGACGACAAGAAAGGGAAACG | CCCTCTAAAATCAATGGCTGCC |
| 117 | Potri.010G006800 | GGCACTTACAACACAGCTGATG | TCAAAACTAGCCATAGCAGCCG |
| 118 | Potri.019G131300 | AAAAACTTCAGGGGTGTCCGTC | TAGTAGGAAAGTTGGTGACGGC |
| 119 | Potri.001G079900 | AGAAGGTGGTGACTAAGGGAAG | TGAGCCACGCATTCTATAAGCC |
| 120 | Potri.015G023200 | CAAACTTCATCACACCGCCTAG | CCGAAGGTAATCTGAACGTGAG |
| 121 | Potri.004G141200 | GTAGAAGCAGTAACGGGAATGG | CTTATTTCTGCCGCCCATTTCC |
| 122 | Potri.010G072300 | GGTTAGGCACATTTGACAGTGC | CCTGCTTCCCAATTTCCTTCTC |
| 123 | Potri.018G038100 | GGCGACGTTTCATTTTCCAACG | CCCACTAAAAATCCCCTCCAAG |
| 124 | Potri.018G047300 | TGCCAGGTTTGCTAATGAGGAG | ATGCTATATTCAGACGGAGGCG |
| 125 | Potri.004G187000 | CACTGAAGGGAGAAACTGCTAC | GCCTCCTCATCAAAGAACAGTG |
| 126 | Potri.006G218200 | GTTGCTGCCAGGTTTGCTAATG | GGAGGGGGATAATAATCACCAC |
| 127 | Potri.006G138800 | GGTTAGGGTCGTATGATTCTGC | CGAGATTCGGATACAGATACGG |
| 128 | Potri.015G136400 | TGATGTTGCTGCTTTGGCGTTG | GTTAACTCAACTCCCAGACTCC |
| 129 | Potri.006G138700 | AGAACCCTCCAAACATAGCAGG | TACTCTGTGGGGTGATTACTCG |
| 130 | Potri.019G015500 | GAGCCCCCCATCAACTCTTTTG | GCCTCGGCAGTATCAAATGTTC |
| 131 | Potri.010G072600 | GCCTATGACAGAGCAGCTTATG | GCAGCTCCTCAAGCAACTTATC |
| 132 | Potri.014G126100 | GTCCCAAAACTCGAACACCAAG | CATTCTGATTACAGCCCAGCAG |
| 133 | Potri.007G046500 | TTTATTTGAGAGGGCCATCGGC | ATGATGAAGGTGGTGGTGGTGG |
| 134 | Potri.017G013700 | CAACTTTCCTTTTTCCGGGGAG | TAACATTCTGCTGCTGCTGCTG |
| 135 | Potri.008G166100 | GGGACATTTGATACGGCAGAAG | CTTCCCCTCTCAAAACTCTCAG |
| 136 | Potri.002G094200 | CAAATCGGCACACCACCAAAAG | GCTGCTCTGTCATAAGCCAAAG |
| 137 | Potri.001G110500 | GCCCTAAAAGGTAATGGTGCAG | TGATGCCAAATACGCACCGATG |
| 138 | Potri.013G045200 | TTATAGAGGGGTTAGAAGGCGG | GACTCGTAGACTTTTTGGGCAG |
| 139 | Potri.019G102200 | TGATGATGATGAACAGGCTGGG | CAGCTTAGCATTCTCTCCTCTC |
| 140 | Potri.014G099900 | ATCTGGCTAGGCTCATTTCCTG | ATCATCATCGGCAACTTCTCCG |
| 141 | Potri.006G104200 | GGAGTTGAAAGCTGTGGAATGG | GCTGGATTCCACAACCTCATTC |
| 142 | Potri.013G135600 | GCCTCCGATTCAGCCTATAAAC | TTCAGCCTAGCATTCTCTCCTC |
| 143 | Potri.001G155700 | CGAAATCCGGCAACCAAAGAAG | CTTTGGACTTGTCTTGCAGCTC |
| 144 | Potri.005G223200 | AATGGCATCAGGGTTTGGCTAG | AGTGTTTCCTCTTCAGTGCCAC |
| 145 | Potri.002G039000 | GAACAGGAAGCCACTCCAAAAG | ACCAGTCTCAAATGTGCCAAGC |
| 146 | Potri.016G053200 | AGCGTTCATGTCATCCATCAGC | TCCACATCTTTCTCTGCTACGG |
| 147 | Potri.005G087200 | GCAGCAAGAGCCTATGATGAAG | TGGATGTAGGAACGGACAGAAG |
| 148 | Potri.002G201600 | CTATGTACCAAACCCCCACTTG | TCCACATCTTTCTCTGCTACGG |
| 149 | Potri.012G134000 | TTAGGTTACGGAGCGGGAAATG | AGGCTGGTATTGGGTAGGAAAG |
| 150 | Potri.014G094500 | GACTTATGACACAGCAGAGGAG | ACACTCATCATCACCACCCTTC |
| 151 | Potri.001G110700 | TGTAGCTGCTTTGGCGTTTAGG | CCTCTTCGTCTATATACCCCAC |
| 152 | Potri.003G033000 | CGCCAAAACAAACTTCCCTGTG | GCTTTTGCCAGACTCCAATGTG |
| 153 | Potri.006G138900 | GAGGAAGCAGCAAGAGCATATG | GATTCCACAATCCTCTCTGCAG |
| 154 | Potri.003G136300 | ACTGCTGAAGAAGCTGCTATGG | GTCTTTCGGGTTTCTCTGCTTG |
| 155 | Potri.011G056900 | CTCACCACCACCATCTTCATTG | GGGGGCTCGTTCAAATCTATTG |
| 156 | Potri.002G172200 | ATTCAAGCAGCAGCAGCAAAGG | CCATCCACAAGAATCCCAACTG |
| 157 | Potri.001G110800 | GGGAACCAAACAAGAAGTCACG | CTTGTTGTTTTGCCGGATGACC |
| 158 | Potri.011G148900 | CTCGCTTCTATTACTCACGGAG | GCCATTCCATGTTTGCTGTTGC |
| 159 | Potri.002G124000 | AGAGGTCTAGGATTTGGCTTGG | TCAACTTTAGCCCCGACTTCAG |
| 160 | Potri.017G053700 | AGAAATCACCAGACACACCACC | CCCATCACTAACACTGCTTCTC |
| 161 | Potri.018G021900 | GCAGCAAGAGCCTATGATGAAG | GCAATGGACTTTTCTCTGGCTG |
| 162 | Potri.001G163700 | TCGTGGCAAGAAAGCTAAGGTG | ATAGGAGCACTGTCAGATGGAG |
| 163 | Potri.014G046700 | TTACTTGCCACCACCAAAAGGG | CGAGCCAATCAAATGTGGGAAG |
| 164 | Potri.014G046600 | TATGACCACGACGACAGAAGAG | TCCTCGCTCCATTCTTCTTAGG |
| 165 | Potri.001G157100 | ATCAATGTCCCTCCCTCTCATG | AACCCCGAAGTTGACGAATCAG |
| 166 | Potri.011G061700 | TAGCAAAGAAGCATTACCGGGG | GATATAGCAGCCACCACTTCAG |
| 167 | Potri.001G079600 | TAGCACCCAAGAAACCTGTAGC | GTACCTAACCAAACCCGACTAC |
| 168 | Potri.001G094800 | GGCTGAGATTAGAGATCCCTTG | CTAAACCGGAGCACTGAGATTG |
| 169 | Potri.001G313500 | GAAAGAAAACACGAAAGGGGGG | GCACCTCTAAGTCTTCTAGCAG |
| 170 | Potri.008G120100 | GAATCCAGTGACCAAAGTGAGG | CTGGGGAAGTATGCGAAACTAC |
| 171 | Potri.016G018600 | GCTGTTGCTTATGATGTGGCAG | CGACCGTTGGCGTAATTTCTTG |
| 172 | Potri.006G069400 | CAACCTTCACTCACAACACCAC | GGCTCTCATCTCTCTTTCCAAG |
| 173 | Potri.001G397200 | GCTTATTCCTATTCCAACGCGC | CGTCGAAAAAGAAAACTGGGCG |
| 174 | Potri.006G156600 | CAACTCAGCCATCCTCAACTTC | TGTTGAAGATGAGGAGGAGGAG |
| 175 | Potri.007G076800 | GAACACAATGAAACCCGCAGAG | GACGTTTTCTGGGAAGTTGAGC |
