# Supplementary material for: Expression Pattern of ERF Gene Family under Multiple Abiotic Stresses in Populus simonii × P. nigra
Source: Front Plant Sci. 2017 Feb 20;8:181. doi: 10.3389/fpls.2017.00181 (PMC5316532; doi:10.3389/fpls.2017.00181)
Supplement: Supplementary file 5 [file Table_2.doc]

Supplemental Table S2 DEGs, significantly (Corrected P-value≤0.05) enriched GO terms and pathways of the DEGs under the four stresses

|  | Classification | NaCl | KCl | CdCl_2_ | PEG |
| --- | --- | --- | --- | --- | --- |
| DEGs (FDR≤0.05, FC>2) | Ups | 159 | 228 | 234 | 203 |
|  | Downs | 245 | 329 | 419 | 381 |
|  | Total | 404 | 557 | 653 | 584 |
| GO terms (Groups) | Cellular components | 4 | 4 | 1 | 1 |
|  | Molecular functions | 13 | 17 | 16 | 14 |
|  | Biological processes | 16 | 21 | 33 | 34 |
|  | Total | 33 | 42 | 50 | 49 |
| Pathways (DEGs/percentage) | Phenylpropanoid biosynthesis | 13 (30.95%) | 17  (29.82%) | 18  (24.66%) | 17  (32.08%) |
|  | Phenylalanine metabolism | 10  (23.81%) | 12  (21.05%) | 14  (19.18%) | 13  (24.53%) |
|  | Starch and sucrose metabolism | 5  (11.9%) | 10  (17.54%) | 14  (19.18%) | 9  (16.98%) |
|  | Plant hormone signal transduction | 0 | 7  (12.28%) | 8  (10.96%) | 7  (13.21%) |
|  | Others | 14 | 11 | 19 | 7 |
|  | Total | 42 | 57 | 73 | 53 |
