# Supplementary material for: Expression Pattern of ERF Gene Family under Multiple Abiotic Stresses in Populus simonii × P. nigra
Source: Front Plant Sci. 2017 Feb 20;8:181. doi: 10.3389/fpls.2017.00181 (PMC5316532; doi:10.3389/fpls.2017.00181)
Supplement: Supplementary file 6 [file Table_3.doc]

Supplemental Table S3 The number of Up/down-regulated ERF genes in different log ratio regions under the four stresses

|  | log ratio | NaCl | KCl | CdCl_2_ | PEG |
| --- | --- | --- | --- | --- | --- |
| Up-regulated | 0-1 | 18 | 16 | 15 | 11 |
|  | 1-2 | 7 | 6 | 6 | 9 |
|  | 2-5 | 11 | 6 | 8 | 7 |
|  | >5 | 4 | 4 | 1 | 3 |
|  | Total | 40 | 32 | 30 | 30 |
| Down-regulated | 0-1 | 8 | 15 | 14 | 19 |
|  | 1-2 | 12 | 10 | 9 | 6 |
|  | 2-5 | 11 | 14 | 15 | 12 |
|  | >5 | 9 | 6 | 8 | 10 |
|  | Total | 40 | 45 | 46 | 47 |
| Total | Total | 80 | 77 | 76 | 77 |
