# Supplementary material for: Expression Pattern of ERF Gene Family under Multiple Abiotic Stresses in Populus simonii × P. nigra
Source: Front Plant Sci. 2017 Feb 20;8:181. doi: 10.3389/fpls.2017.00181 (PMC5316532; doi:10.3389/fpls.2017.00181)
Supplement: Supplementary file 7 [file Table_4.doc]

Supplemental Table S4 ERF genes (|log2Ratio| >2) shared by the four stresses

|  | Gene ID | Common name | Gene ID in Arabidopsis | NaCl (log2Ratio) | KCl (log2Ratio) | CdCl_2_  ( log2Ratio) | PEG  (log2Ratio) |
| --- | --- | --- | --- | --- | --- | --- | --- |
| Up-regulated | Potri.001G092400.1 |  | AT1G64380.1 | 17.00 | 16.59 | 16.49 | 16.47 |
|  | Potri.005G195000.1 | ERF76 | AT5G50080.1 | 6.74 | 6.10 | 4.30 | 4.34 |
|  | Potri.018G038100.1 |  | AT5G21960.1 | 6.17 | 5.66 | 4.53 | 5.58 |
|  | Potri.004G051700.1 | ERF24 | AT5G47220.1 | 4.87 | 5.15 | 4.92 | 5.13 |
|  | Potri.006G138900.1 | DREB38 | AT5G21960.1 | 4.38 | 3.81 | 3.34 | 4.48 |
|  | Potri.011G061700.1 |  | AT3G23240.1 | 4.22 | 3.97 | 3.80 | 3.95 |
|  | Potri.002G039100.1 | ERF31 | AT3G23240.1 | 3.06 | 2.97 | 2.88 | 3.26 |
|  | Potri.003G139300.1 | DREB14 | AT1G64380.1 | 2.49 | 3.14 | 4.28 | 4.03 |
| Down-regulated | Potri.005G223300.1 | ERF27 | AT3G23240.1 | -16.185 | -16.185 | -16.185 | -16.185 |
|  | Potri.006G238600.1 | DREB80 | AT5G11590.1 | -15.94 | -3.97 | -5.48 | -5.59 |
|  | Potri.T050600.1 | ERF29 | AT3G23240.1 | -15.67 | -15.67 | -15.67 | -15.67 |
|  | Potri.001G094800.1 | ERF87 | AT4G23750.2 | -15.48 | -3.97 | -3.97 | -2.75 |
|  | Potri.019G088000.1 | ERF62 | AT1G12890.1 | -15.43 | -15.43 | -15.43 | -15.43 |
|  | Potri.018G043900.1 | DREB81 | AT5G11590.1 | -7.95 | -5.18 | -5.95 | -18.40 |
|  | Potri.004G047500.1 | ERF43 | AT1G28360.1 | -6.83 | -5.44 | -6.81 | -5.24 |
|  | Potri.006G261200.1 | DREB25/26 | AT5G25190.1 | -6.29 | -6.73 | -8.31 | -5.98 |
|  | Potri.001G187500.1 | DREB77 | AT5G11590.1 | -5.22 | -4.83 | -3.22 | -6.81 |
|  | Potri.019G067400.1 |  | AT5G25810.1 | -4.51 | -2.92 | -2.87 | -4.51 |
|  | Potri.003G151000.1 | ERF2 | AT5G07580.1 | -4.44 | -4.21 | -2.37 | -3.60 |
|  | Potri.008G210900.1 | ERF35 | AT3G16770.1 | -4.34 | -4.13 | -3.56 | -3.79 |
|  | Potri.013G158500.1 |  | AT4G27950.1 | -3.58 | -3.63 | -2.28 | -3.03 |
|  | Potri.018G085700.1 | DREB78 | AT5G11590.1 | -3.07 | -2.56 | -2.10 | -4.07 |
|  | Potri.002G201600.1 | ERF36 | AT3G16770.1 | -3.05 | -3.03 | -3.20 | -4.06 |
|  | Potri.001G154200.1 |  | AT4G17490.1 | -2.75 | -3.40 | -3.56 | -2.67 |
|  | Potri.001G048200.1 |  | AT5G25190.1 | -2.65 | -3.70 | -3.07 | -15.30 |
