# Supplementary material for: Expression Pattern of ERF Gene Family under Multiple Abiotic Stresses in Populus simonii × P. nigra
Source: Front Plant Sci. 2017 Feb 20;8:181. doi: 10.3389/fpls.2017.00181 (PMC5316532; doi:10.3389/fpls.2017.00181)
Supplement: Supplementary file 8 [file Table_5.doc]

Supplemental Table S5 Morphological variation between transgenic poplar and wild type poplar

|  | WT | T-1 | T-2 | T-3 | T-4 | T-5 |
| --- | --- | --- | --- | --- | --- | --- |
| Root number | 9.20±0.09 | 9.20±0.11 | 8.80±0.31 | 8.20±0.09 | 8.10±0.09 | 8.20±1.06 |
| Root length/cm | 6.35±0.39 | 8.20±0.56 | 9.60±0.39 | 8.80±0.25 | 8.55±0.53 | 8.80±0.03 |
| Long axis /μm | 21.98±1.56 | 26.84±0.69 | 28.47±2.58 | 28.05±0.97 | 30.10±2.43 | 27.07±1.27 |
| Short axis /μm | 15.40±0.84 | 16.46±0.78 | 16.96±1.98 | 19.40±0.83 | 17.81±1.41 | 18.82±0.82 |
| Size/μm^2^ | 266.11±27.82 | 346.70±16.51 | 381.81±74.84 | 426.99±12.10 | 420.77±45.46 | 400.38±32.96 |
| Stomata frequency | 4 | 5 | 6 | 6 | 5.5 | 5.5 |
